# "Zombie virus" like pyroptosis: Extracellular vesicles spread pyroptosis by transferring functional N-GSDMD pore

**DOI:** 10.1515/jtim-2026-0003

**Published:** 2026-02-13

**Authors:** Yihang Zhang, Shumei Jin, Yunfen Tian, Jialong Qi

**Affiliations:** Yunnan Digestive Endoscopy Clinical Medical Center, Department of Gastroenterology, The First People's Hospital of Yunnan Province, Kunming, Yunnan Province, China; West China School of Medicine, West China Hospital, Sichuan University, Chengdu, Sichuan Province, China; Yunnan Institute of Food and Drug Supervision and Control, Medical Products Administration of Yunnan Province, Kunming, Yunnan Province, China; Department of pediatrics, The First People's Hospital of Yunnan Province, Kunming, Yunnan Province, China; Yunnan Provincial Key Laboratory of Clinical Virology, The First People's Hospital of Yunnan Province, Kunming, Yunnan Province, China; School of Medicine, Kunming University of Science and Technology, Affiliated by The First People's Hospital of Yunnan Province, Kunming, Yunnan Province, China; Yunnan Provincial Key Laboratory of Birth Defects and Genetic Diseases, First People's Hospital of Yunnan Province, Kunming, Yunnan Province, China; Yunnan Province Clinical Research Center for Senile diseases, First People's Hospital of Yunnan Province. Kunming, Yunnan Province, China

Recent studies highlight pyroptosis as a critical immune defense mechanism, yet its dysregulation also drives pathological inflammation. In a study published in Cell, Rathinam and colleagues revealed that extracellular vesicles (EVs) released by pyroptotic cells contain activated N-terminal gasdermin D (GSDMD-NT), which transfers pre-formed pores to neighboring cells and induces their death independently of inflammasome activation. This unexpected mechanism of pyroptosis propagation raises new questions about its role in inflammatory diseases such as sepsis and autoimmunity.

Pyroptosis is a programmed inflammatory and lytic form of cell death driven by an activated gasdermin family member.^[1]^ Upon pathogen infection, the canonical inflammasome pathway activates caspase-1, whereas the noncanonical pathway engages caspase-4/5/11 to cleave gasdermin D (GSDMD), liberating its pore-forming N-terminal fragment.^[2]^ The resulting GSDMD-NT oligomers perforate the plasma membrane, leading to cellular lysis and the release of interleukin-1β (IL-1β), interleukin-18 (IL-18), and other proinflammatory mediators. Recent studies have also revealed inflammasome-independent mechanisms, such as palmitoylation-mediated membrane targeting of GSDMD, which can initiate pyroptosis without proteolytic cleavage^[3]^ (Figure 1).

**Figure 1:**
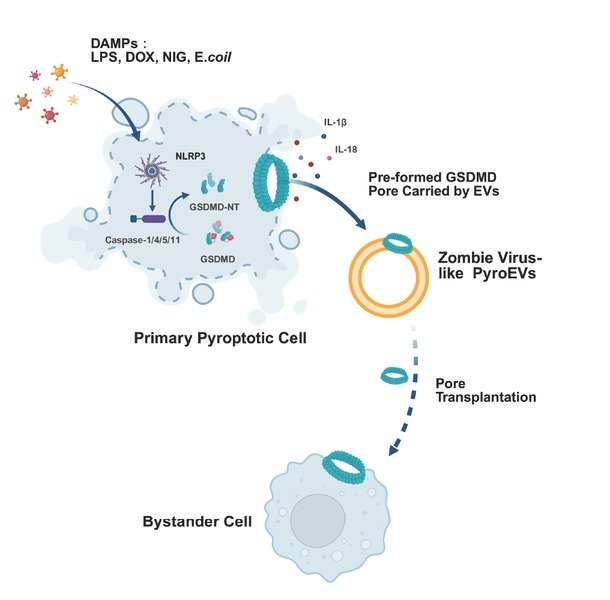
Mechanism of Pore Transplantation *via* Pyroptotic Extracellular Vesicles (PyroEVs) Damage-associated molecular patterns (DAMPs) such as LPS, doxorubicin (DOX), nigericin (NIG) and *E. coli* (*Escherichia coli*) activate the NLRP3 inflammasome in primary pyroptotic cells, leading to caspase-1/4/5/11 activation and cleavage of GSDMD. The activated GSDMD-NT forms membrane pores, facilitating the release of IL-1β, IL-18, and HMGB1. Meanwhile, PyroEVs harboring preassembled GSDMD pores mediate intercellular transfer of these cytotoxic structures to adjacent cells through a zombie-like transmission mechanism, thereby disseminating pyroptotic signaling cascades beyond the primary site of cellular activation.

A concise overview of the classical, nonclassical, and alternative pyroptotic pathways—including their stimuli, core sensors, caspases, and downstream effectors—is summarized in Supplementary Table 1 for clarity. While pyroptosis is essential for host defense, its dysregulated activation can lead to uncontrolled inflammation and bystander cell death even in cells lacking inflammasome-mediated pathogen sensing.^[4]^ The mechanisms underlying this bystander cell death remain unclear, and the impact of pyroptosis in disease pathogenesis is not well understood.

Interestingly, some cells did not receive a direct death signal but instead perished due to harmful factors released by their dying pyroptotic neighbors. In experiments by Rathinam *et al*.^[5]^ stimulation with nigericin—a recognized pyroptosis inducer—resulted in the death of cells resistant to the stimulus after the demise of the more susceptible cells. Furthermore, direct induction of GSDMD-NT expression to initiate pyroptosis led to the death of neighboring cells that were not originally activated when co-cultured. Supporting these observations, experiments in three mouse models demonstrated that following the pyroptosis of target cells, bystander cells also died *in vivo*.

To determine whether pyroptosis requires direct cell contact, the authors used a transwell system to separate pyroptotic cells from naive cells. Remarkably, bystander cells still underwent pyroptosis despite physical segregation. This contact-independent propagation was further confirmed by inducing pyroptosis in naïve cells using pyroptotic cell supernatants. Using size-exclusion chromatography (SEC), the authors identified EVs as the component responsible for cell-lytic activity. They further demonstrated that EVs derived from pyroptotic cell supernatants, referred to as pyroptotic extracellular vesicles (pyroEVs), can induce bystander cell death both *in vitro* and *in vivo* (Figure 1).

To elucidate the mechanism by which EVs mediate the spread of pyroptosis, the authors exposed GSDMD-deficient cells to pyroEVs, inducing cell death. This finding suggests that bystander cell death occurs independently of inflammasome-activating damage-associated molecular patterns (DAMPs) and the recipient cell’s inflammasome machinery. To determine whether pyroEVs carry active GSDMD, the authors used immunoblotting and imaging flow cytometry, confirming the presence of GSDMD-NT in EVs released from pyroptotic cells. Furthermore, confocal microscopy confirmed GSDMD-NT signals are primarily localized on the plasma membrane of bystander cells, indicating that pyroEVs transfer GSDMD-NT to recipient cells, ultimately triggering their death.

Super-resolution DNA-PAINT imaging revealed that GSDMD-NT pores are pre-assembled on PyroEV surfaces, and detergent extraction confirmed their structural integrity. These pores are stabilized on EV membranes *via* lipid-protein interactions within cholesterol- and phosphatidylserine-enriched microdomains, which provide favorable curvature and electrostatic environments for oligomer stability.^[6]^ PyroEVs can fuse with recipient cell membranes through canonical EV uptake pathways, including tetraspanin-dependent fusion and SNARE-mediated docking,^[7]^ enabling the direct delivery of preformed GSDMD-NT pores to bystander cells.

To test the requirement for new pore assembly, disulfiram—a covalent inhibitor of the Cys 191 residue of GSDMD—was applied. When administered after pyroEV isolation, disulfiram did not block liposome leakage induced by GSDMD-NT-containing EVs, confirming that pores were pre-assembled within EV membranes. In contrast, disulfiram treatment before pyroptosis induction suppressed both GSDMD cleavage and subsequent EV-mediated liposome leakage, supporting the direct pore-transfer mechanism.

In addition to GSDMD pores, subsets of pyroEVs may carry inflammasome components such as active caspase-1 and ASC. While these proteins can activate inflammasomes in recipient cells, the present data indicate that pyroEV-mediated membrane leakage and bystander cell death occur independently of caspase-1, emphasizing that direct pore transfer is the dominant pathway in this context. Collectively, these findings provide a mechanistic basis for the rapid, contact-independent propagation of pyroptosis *via* pore transplantation.

It is widely recognized that eukaryotic cells secrete EVs, which facilitate intercellular communication by transferring bioactive molecules. In targeted drug delivery, EVs act as carriers for therapeutic agents such as siRNA or proteins, enabling precise modulation of cellular functions.^[8]^ This suggests a potential role in the propagation of pyroptosis, where EVs may mediate the transfer of pyroptosis-related components between cells. Bacteria also secrete EVs, known as outer membrane vesicles (OMVs), which function as “biological missiles” in infection and immune regulation.^[9]^ OMVs deliver virulence factors such as LPS and bacterial toxins to recipient cells, activating inflammasomes and triggering pyroptosis. These findings suggest that both eukaryotic and prokaryotic organisms can propagate pyroptosis through EV-mediated transfer of key components, revealing a conserved mechanism of inflammatory cell death.

Studies have shown that EVs derived from pyroptotic cells can further propagate pyroptosis by delivering inflammatory mediators, such as LPS and apoptosis-associated speck-like protein containing a CARD (ASC).^[10]^ These molecules activate the NOD-like receptor family pyrin domain-containing 3 (NLRP3) inflammasome assembly in recipient cells, enhancing GSDMD cleavage and amplifying pyroptotic signaling, ultimately exacerbating systemic inflammation. However, pyroEVs do not always promote inflammation. While pyroEVs can amplify inflammatory signaling, their effects are context-dependent. In sepsis, circulating EVs enriched in inflammasome components and GSDMD have been associated with increased cytokine release and multi-organ dysfunction, potentially contributing to cytokine storm syndrome.^[11, 12, 13]^ Conversely, ASC-positive EVs derived from mesenchymal stem cells have been reported to attenuate hyperinflammation by modulating B cell responses and suppressing TLR4-mediated pyroptosis.^[14]^ Similarly, mesenchymal stem cell–derived exosomes have been shown to exert potent regenerative and signaling-modulatory effects *via* activation of the RAS/ERK pathway, further supporting the immunoregulatory and reparative potential of stem cell–derived EVs.^[15]^ These findings indicate that the cellular origin and cargo composition of pyroEVs determine whether they exacerbate or mitigate disease. Importantly, recent preclinical studies targeting EV biogenesis pathways—such as nSMase2 inhibition by GW4869 or Rab27a suppression—demonstrate attenuation of systemic inflammation and tissue injury in sepsis and autoimmune models.^[14,16]^ Circulating inflammasome-positive EVs have also been proposed as biomarkers correlating with disease severity, highlighting their combined diagnostic and therapeutic potential.

Unlike classical intercellular communication through soluble cytokines (*e.g*., IL-1β, IL-18) or DAMPs, which activate recipient cells *via* receptor-mediated signaling, vesicle-mediated GSDMD pore transfer delivers preformed structural death-executing components directly into the plasma membrane. This “structural” mode of communication is distinct from chemical signaling and may act synergistically with cytokine and DAMP release to amplify inflammatory responses. Incorporating this perspective underscores both the novelty and broader mechanistic implications of the pore transfer pathway.

Mechanistically, Rathinam’s groundbreaking work^[5]^ redefines pyroptosis propagation: While conventional models emphasize EV-mediated inflammasome activation in bystander cells, their study reveals a direct transmembrane delivery system. Instead of relying on intracellular signaling, EVs in this model transfer active pyroptotic components directly. This “pore transplantation” mechanism achieves faster pyroptosis propagation compared to classical inflammasome-dependent pathways, fundamentally altering our understanding of inflammatory signal amplification in tissue microenvironments.

Collectively, Rathinam *et al*.’s findings position EVs as active carriers of cytolytic machinery and introduce the concept of pore transplantation as a rapid, contact-independent mode of pyroptosis propagation. Nonetheless, several critical questions remain: how are GSDMD pores selectively loaded and stabilized on EV membranes; what molecular determinants govern pyroEV-recipient membrane fusion; and to what extent do inflammasome proteins packaged within pyroEVs cooperate with pore transfer to shape pathological outcomes? Addressing these questions—together with translational studies that evaluate EV biogenesis inhibitors and EV-based biomarkers—will be essential to convert this mechanistic insight into clinically actionable strategies for inflammatory diseases.

## Supplementary Material

Supplementary Material Details
