# Supplementary material for: "Zombie virus" like pyroptosis: Extracellular vesicles spread pyroptosis by transferring functional N-GSDMD pore
Source: J Transl Int Med. 2026 Feb 13;14(1):10–3. doi: 10.1515/jtim-2026-0003 (PMC12916272; doi:10.1515/jtim-2026-0003)
Supplement: Supplementary file 1 — Supplementary Material Details [file jtim-2026-0003_sm.pdf]

Supplementary materials

Table S1. Classical and nonclassical pyroptosis pathways

Summary of representative pyroptosis pathways, including their canonical and noncanonical forms, apoptotic crosstalk mechanisms, and alternative gasdermin activations[17]. Each pathway is characterized by its distinct triggers, inflammasome involvement, activating caspases, and key gasdermin effectors. While canonical and noncanonical pathways predominantly involve GSDMD-mediated pore formation and IL-1β/IL-18 release, other gasdermins such as GSDME, GSDMB, and GSDMC participate in apoptosis-linked or cytokine-limited pyroptotic responses.

**Pathway**	**Triggers**	**Inflammasome or Activator**	**Caspase (s)**	**Key Gasdermin (s)**	**Main Cytokines**
**Canonical**	PAMPs, DAMPs, ATP, nigericin, *E. coli*, DOX	NLRP3, AIM2, NLRC4, *etc.* + ASC	Caspase-1	GSDMD	IL-1β/IL-18 release, cell lysis
**Noncanonical**	Cytosolic LPS (Gram-negative bacteria)	None (direct LPS sensing)	Caspase-4/5 (human), Caspase-11 (mouse)	GSDMD	IL-1β/IL-18 release (*via* secondary NLRP3), cell lysis
**Apoptosis–Pyroptosis**	Chemotherapy, immune effectors (Granzyme B)	Apoptotic proteases	Caspase-3, others	GSDME (sometimes GSDM)	IL-1β/IL-18 release, pyroptosis, context-dependent tumor effects
**Alternative Gasdermin Activation**	TNF-α, IFN-γ, Caspase-8, Granzyme A	Noncanonical proteases	Caspase-8, Granzyme A	GSDMB, GSDMC	Cell death, cytokine release

DAMPs, Damage-associated molecular patterns; IL-1β, interleukin-1β; GSDMD, gasdermin D.
